# Domestic light at night and breast cancer risk: a prospective analysis of 105 000 UK women in the Generations Study

**DOI:** 10.1038/bjc.2017.359

**Published:** 2018-01-23

**Authors:** Louise E Johns, Michael E Jones, Minouk J Schoemaker, Emily McFadden, Alan Ashworth, Anthony J Swerdlow

**Affiliations:** 1 Division of Genetics and Epidemiology, The Institute of Cancer Research, London SM2 5NG, UK; 2 Division of Breast Cancer Research, The Institute of Cancer Research, London SW3 6JB, UK; 3 Breast Cancer Now Research Centre at The Institute of Cancer Research, London SW3 6JB, UK; 4 Division of Molecular Pathology, The Institute of Cancer Research, London SW7 3RP, UK

**Keywords:** breast cancer, cohort study, light at night, risk

## Abstract

**Background:**

Circadian disruption caused by exposure to light at night (LAN) has been proposed as a risk factor for breast cancer and a reason for secular increases in incidence. Studies to date have largely been ecological or case-control in design and findings have been mixed.

**Methods:**

We investigated the relationship between LAN and breast cancer risk in the UK Generations Study. Bedroom light levels and sleeping patterns at age 20 and at study recruitment were obtained by questionnaire. Analyses were conducted on 105 866 participants with no prior history of breast cancer. During an average of 6.1 years of follow-up, 1775 cases of breast cancer were diagnosed. Cox proportional hazard models were used to calculate hazard ratios (HRs), adjusting for potential confounding factors.

**Results:**

There was no association between LAN level and breast cancer risk overall (highest compared with lowest LAN level at recruitment: HR=1.01, 95% confidence interval (CI): 0.88–1.15), or for invasive (HR=0.98, 95% CI: 0.85–1.13) or *in situ* (HR=0.96, 95% CI: 0.83–1.11) breast cancer, or oestrogen-receptor (ER) positive (HR=0.98, 95% CI: 0.84–1.14); or negative (HR=1.16, 95% CI: 0.82–1.65) tumours separately. The findings did not differ by menopausal status. Adjusting for sleep duration, sleeping at unusual times (non-peak sleep) and history of night work did not affect the results. Night waking with exposure to light, occurring around age 20, was associated with a reduced risk of premenopausal breast cancer (HR for breast cancer overall=0.74, 95% CI: 0.55–0.99; HR for ER-positive breast cancer=0.69, 95% CI: 0.49–0.97).

**Conclusions:**

In this prospective cohort analysis of LAN, there was no evidence that LAN exposure increased the risk of subsequent breast cancer, although the suggestion of a lower breast cancer risk in pre-menopausal women with a history of night waking in their twenties may warrant further investigation.

Breast cancer is the most common cancer diagnosed in women worldwide and incidence continues to rise. Internationally, the highest rates are seen in economically developed countries (DeSantis *et al*, 2015). This pattern of incidence, coupled with observations from migration studies (Ziegler *et al*, 1993; Deapen *et al*, 2002), is consistent with a strong role for lifestyle and environmental factors influencing breast cancer risk.

In 1978 Cohen *et al* (1978) suggested that disruption to circadian rhythm could result in higher circulating oestrogen levels and thereby increase the risk of breast cancer, and in 1987 Stevens (Stevens, 1987) proposed that secular trends in breast cancer incidence might be explained by increasing exposure to artificial light. The hypothesised mechanism is via melatonin, a hormone secreted by the pineal gland in response to decreases in ambient light (Huether, 1993; Brainard *et al*, 2001).

This ‘light at night’ (LAN) theory has stimulated epidemiological investigations. To date, most studies have been either ecological in design, correlating cancer incidence rates in populations with estimates of outdoor ambient LAN (Kloog *et al*, 2008, 2010; Kim *et al*, 2016), or case-control studies, examining risks associated with self-reported measures of indoor LAN (Davis *et al*, 2001; O'Leary *et al*, 2006; Bauer *et al*, 2013). These have disadvantages in that the former do not analyse on an individual basis and the latter have potential for recall and selection biases. Only one cohort study has been published, which found an increased risk in relation to outdoor LAN, measured by satellite, but not indoor LAN, measured by questionnaire (Hurley *et al*, 2014). Overall, epidemiological findings have been inconsistent, and the metrics analysed have varied and not been clearly comparable (O'Leary *et al*, 2006; Bauer *et al*, 2013; Hurley *et al*, 2014).

Here we report a cohort study of the relationship between LAN and subsequent breast cancer risk, based on data from women recruited to the UK Generations Study.

## Methods

### Study population

This analysis is based on the Generations Study (GS), a cohort study of >113 000 women from the UK, recruited at ages ⩾16 years since 2003. Recruitment involved a baseline postal questionnaire about established and potential breast cancer risk factors, and donation of a blood sample. Participants are followed up approximately every 3 years, by postal or online questionnaires, to obtain updated risk factor and outcome information (further detail in Swerdlow *et al*, 2011). The study was undertaken with informed consent and ethics approval from the South East Multi-Centre Research Ethics Committee.

Cancers occurring in the cohort were identified from recruitment and follow-up questionnaires, spontaneous reports to the study centre, and ‘flagging’ at the National Health Service Central Registers, virtually complete registers of the population of the country, which notify cancer registrations, deaths and emigrations in study subjects to authorised medical researchers. Confirmation of self-reported cancer diagnoses was obtained from medical records.

### Exposure assessment

Information on LAN and sleep patterns in GS participants was obtained at recruitment. Women were asked to report their level of exposure to LAN over the year prior to recruitment and at age 20 years, in the room in which they slept, in the categories; ‘light enough to read’ ‘light enough to see across the room, but not read’ ‘light enough to see your hand in front of you, but not to see across the room’ and ‘too dark to see your hand, or you wear a mask’.

### Covariate information

Detailed information on established breast cancer risk factors was collected by the GS at recruitment and was updated, where applicable, at follow-up rounds (Swerdlow *et al*, 2011). Information on history of night shift work (defined as work between 2200 and 0700 hours) obtained at baseline was used to derive a dichotomous variable for ever/never night work during the 10 years before recruitment. The average number of times GS participants woke during the night and put on a light or entered a bright room over the year prior to recruitment and at age 20 years was collected at baseline. Non-peak sleep (Davis *et al*, 2001) was assessed as going to sleep at or after 0200 hours or rising for the day at or before 0100 hours. GS participants were also asked about average sleep duration, which was used to derive a dichotomous variable <7 h *vs* ⩾7 h sleep per night, based on median sleep in the GS cohort and thresholds used in other studies (Verkasalo *et al*, 2005; Pinheiro *et al*, 2006; Kakizaki *et al*, 2008).

### Statistical analysis

The current analytic cohort is based on all women who were recruited to the study during June 2003 to June 2012 inclusive, and who had not previously been diagnosed with breast cancer. Follow-up for breast cancer started at the date of receipt of the recruitment questionnaire and ended on the earliest of: breast cancer, death, date of mailing for follow-up questionnaires, or if the follow-up questionnaire was not returned and the woman was covered by ‘flagging’, the earliest of the date the individual’s ‘flagging’ coverage ended (i.e., when she was removed from the NHS Central Register), or the date after which ‘flagging’ notification was not yet complete (taken to be 1 March 2014). If the follow-up questionnaire was not returned and the woman was not covered by ‘flagging’ (<1.2% of the cohort), the follow-up was truncated at the date of her last returned questionnaire.

Only a small proportion of women reported LAN in the lightest category (‘light enough to read’ 0.96% for the year before recruitment and 1.92% at age 20). This group was therefore combined with the adjacent group (‘light enough to see across the room, but not read’). Cox proportional hazards regression (Cox, 1972) using attained age as the implicit timescale was used to estimate hazard ratios (HRs) and 95% confidence intervals (CIs) for breast cancer risk in relation to LAN exposure. To control for potential confounding by other breast cancer risk factors, we adjusted for year of birth, history of benign breast disease, history of breast cancer in a first-degree relative, socioeconomic score, age at menarche, age at first birth, parity, duration of breastfeeding, oral contraceptive (OC) use before menopause, hormone replacement therapy (HRT) use, menopausal status, age at menopause, pre-menopausal and post-menopausal body mass index, alcohol consumption, smoking and leisure time physical activity (in metabolic equivalents, METS, hours per week), with time-varying data incorporated for age at first birth, parity, menopausal status, age at menopause, OC use and HRT use. Socioeconomic score was based on place of residence (Acorn scores; Solutions CI, 2002).

Results are presented by oestrogen-receptor (ER) status of breast cancer and menopausal status during follow-up. Age at menopause was assumed to be 50 years for women whose menopausal status or age at menopause was not known. Analyses presented in the tables are for invasive plus *in situ* breast cancer combined, but we also conducted analyses separately for invasive and *in situ* disease and for broad morphological groups (ductal, lobular, other/not known), which are reported in the text. Analyses were also adjusted for ever night shift work, sleep duration, and non-peak sleep to investigate their potential impact.

All statistical tests were performed using Stata/IC version 14.0 (StataCorp, 2015) and all reported *P*-values were two-sided.

## Results

Of the 113 207 women recruited to the GS during June 2003 – June 2012, 6581 were excluded from analysis because of prior breast cancer, 14 due to prior bilateral mastectomy and 746 due to missing LAN information, leaving 105 866 women included in the analysis. Their mean age at recruitment was 46.5 years (range 16–102 years) and mean follow-up was 6.1 years (standard deviation=1.0). Follow-up by questionnaire (96.3%) or ‘flagging’ (1.7%) was complete for 98% of women, 0.8% (*n*=809) had died and 1.2% (*n*=1323) were lost to follow-up (e.g., by emigration). During follow-up, 1775 breast cancers (1503 invasive and 272 *in situ*) were diagnosed. ER status was ascertained for 99% of invasive and 58% of *in situ* cases; the latter was lower because hormone receptor assays are often not undertaken for *in situ* lesions in the UK. Histology was ascertained for 95% of cases. Further details of breast cancer cases are described in Supplementary Table 1.

Characteristics of the cohort are shown in Table 1. Women from earlier birth cohorts tended to report lower LAN levels, but overall, ‘medium’ levels of LAN were the most common, reported by 49% of participants for the year before recruitment and 47% of participants for age 20. Overall, 97% (*n*=102 972) of participants in this analysis reported LAN information at age 20. A greater proportion of women reported waking at night and turning on lights or going into a bright room during the year before recruitment than at age 20 (37% *vs* 10%, respectively, *P*<0.001). Seventeen percent of participants reported a history of night shift work during the 10 years before recruitment (we did not have consistent information on shift work before that).

There was no statistically significant association between breast cancer risk and LAN level in the year before recruitment or at age 20, when adjusted for age and year of birth. The HR for the highest LAN compared with the lowest LAN category at recruitment was 1.01 (95% CI: 0.88–1.15; Table 2) and for LAN at age 20 was 1.00 (95% CI: 0.88–1.15; Table 3). For pre-menopausal breast cancer, the adjusted HR for the highest *vs* lowest LAN level at recruitment was 1.00 (95% CI: 0.81–1.24) and at age 20 was 0.91 (95% CI: 0.73–1.13). Similarly, there was no significant association between LAN and breast cancer risk when the analysis was limited to post-menopausal follow-up (Table 2). Analyses in relation to ER sub-type showed no association: for LAN at recruitment, the HR for ER-positive breast cancer was 0.98 (95% CI: 0.84–1.14) and for ER-negative disease was 1.16 (95% CI: 0.82–1.65).

When analyses were repeated to investigate the risk of invasive and *in situ* breast cancer separately, results were very similar to those for breast cancer overall: HR=0.98 (95% CI: 0.85–1.13) for invasive and HR=0.96 (95% CI: 0.83–1.11) for *in situ* breast cancer for the highest *vs* lowest LAN level at recruitment (not in Table). Similarly, there was no association between LAN and the risk of different morphological subtypes of breast cancer: for the highest *vs* lowest level LAN at recruitment, ductal HR=1.00 (95% CI: 0.86–1.16), lobular HR=0.92 (95% CI: 0.66–1.28) and other types HR=1.41 (95% CI: 0.85–2.35) (not in Table).

There was no relationship between reported night waking with exposure to light in the year before recruitment and risk of breast cancer (HR=1.01, 95% CI: 0.92–1.12; Table 4). Similarly, there was no association between this exposure and breast cancer risk in pre- or post-menopausal women and results did not differ by ER status of breast cancer. For night waking with light exposure at age 20, however, there was a reduced risk of pre-menopausal breast cancer (HR=0.74, 95% CI: 0.55–0.99, *P*=0.04; Table 5), with a reduced risk of ER-positive (HR=0.69, 95% CI: 0.49–0.97, *P*=0.03), but not ER-negative (HR=0.91, 95% CI: 0.45–1.82) cancers and no effect for post-menopausal breast cancer.

There was no impact on our results when analyses comparing the highest LAN *vs* lowest LAN categories at recruitment were adjusted for history of night shift work in the 10 years before recruitment (HR=1.01, 95% CI: 0.88–1.15), duration of sleep (HR=1.01, 95% CI: 0.89–1.15) or non-peak sleep (HR=1.01, 95% CI: 0.88–1.15) (not in Table).

## Discussion

It has been hypothesised that suppression of nocturnal pineal melatonin production in response to LAN might explain the rises in breast cancer rates that have accompanied industrialisation and electrification in westernised countries (Stevens, 1987). Exposure to artificial light during the night can disrupt the circadian rhythm and reduce the normal nocturnal rise in melatonin (Stevens, 1987; Stevens and Rea, 2001; Claustrat *et al*, 2005; Stevens *et al*, 2007; Straif *et al*, 2007), leading to an increase in circulating oestrogen levels (Cohen *et al*, 1978; Cos and Sanchez-Barcelo, 2000) and suppression of tumour anti-proliferative processes, which might increase breast cancer risk (Stevens, 1987; Stevens and Rea, 2001; Hill *et al*, 2015).

Clearly, if circadian disruption of melatonin plays a substantial role in the aetiology of breast cancer, it is of major public health importance. Following early epidemiologic studies (Davis *et al*, 2001; Hansen, 2001; Schernhammer *et al*, 2001; O'Leary *et al*, 2006), the World Health Organisation has designated night shift work involving LAN-induced circadian/melatonin disruption as a probable carcinogen (class 2a) and risk factor for the development of breast cancer (Straif *et al*, 2007). In Denmark this led to a change to occupational compensation law (Wise, 2009).

In our analysis of over 105 000 UK women, we found no evidence of a relationship between self-reported level of domestic exposure to LAN and subsequent raised risk of breast cancer. Published ecological studies using satellite data to derive LAN exposure in Israel, South Korea and worldwide have tended to show high light levels associated with a 30–70% increased risk of breast cancer (Kloog *et al*, 2008; 2010; Kim *et al*, 2016). Findings from case-control studies conducted in a number of countries have been more mixed. Using self-reported bedroom LAN data, several studies found statistically non-significant increased risks ranging from 10 to 50% (Davis *et al*, 2001; O'Leary *et al*, 2006; Li *et al*, 2010; Keshet-Sitton *et al*, 2016). Statistically significant increased risks of 10–20% have, however, been reported by case-control studies of LAN exposure in Israel (Kloog *et al*, 2011) and the US (Bauer *et al*, 2013). Ecological and case-control study designs have disadvantages, however: potential confounding by other factors affecting breast cancer risk, inability to link exposure directly to individual outcome in ecological studies, and potential selection and recall biases in case-control studies. Cohort studies provide a mechanism for avoiding these deficiencies. To the best of our knowledge, the only previously published cohort study was that by Hurley *et al* (2014) among teachers in California. That analysis of 106 731 female Californian teachers found an increased risk of breast cancer for women living in areas with the highest quintile of estimated outdoor LAN exposure as assessed from satellite data, but no effect of indoor LAN assessed from questionnaire responses incorporating duration of use.

When we examined LAN effects in pre- and post-menopausal women separately, we found no difference in breast cancer risk in relation to bedroom light level by menopausal status. A small number of published studies investigated risk by menopausal status. In the only cohort study (Hurley *et al*, 2014), there was a more pronounced risk of breast cancer associated with outdoor LAN in pre-menopausal than post-menopausal women, while in case-control studies, Li *et al* (2010) observed a non-significant increased risk in post-menopausal women only and O'Leary *et al* (2006) found similar LAN effects irrespective of menopausal status.

In addition to LAN, a range of other exposure variables have been used to assess potential disruption to circadian rhythm, such as duration of sleep, non-peak sleep, night waking with exposure to light, and night shift work (Davis *et al*, 2001; Schernhammer *et al*, 2001; O'Leary *et al*, 2006; Straif *et al*, 2007; Li *et al*, 2010; Qian *et al*, 2015; Keshet-Sitton *et al*, 2016). There is a potential interplay between these variables and LAN. For example, people who have difficulty sleeping may spend more time awake with a light on during the night. Similarly, non-peak sleep may result in increased exposure to artificial light during the hours of natural darkness. In our study, there was still no association between LAN exposure and risk of breast cancer after adjustment for average sleep duration and nonpeak sleep. Likewise, after adjustment for history of night shift work in the 10 years before recruitment, we found no association between bedroom LAN and risk of breast cancer. Studies have shown that intermittent nocturnal light exposure of sufficient intensity lowers melatonin levels (Bojkowski *et al*, 1987; Brzezinski, 1997; Travlos *et al*, 2001). In our study, we found self-reported night waking with light exposure at age 20 was associated with a decreased risk of pre-menopausal breast cancer, particularly ER-positive cancer. Epidemiological investigations of night waking that leads to light exposure have had mixed findings: one study (Davis *et al*, 2001) found no relation, while another (O'Leary *et al*, 2006) found a significant 65% increase in breast cancer risk.

Major strengths of our study are its prospective design, large study population size, comprehensive assessment of breast cancer risk factors and very high follow-up rates. The detailed information on established breast cancer risk factors available within the GS allowed us to adjust for a wide range of potentially confounding factors in our analyses. The Californian Teachers cohort study and most case-control studies have adjusted for the major recognised breast cancer risk factors, but few ecological studies, with the exception of Kloog *et al* (2008) have been adjusted for potential confounders. Our study also has the advantage of having information on reported LAN at age 20.

LAN reflects the degree of exposure to internal lights left on at night, plus both the extent of external light (natural and artificial) and the extent of window covering blocking light entry. The LAN measure we used takes in all of these factors, whereas a residential address only gives potential information on external light. Thus a woman in a dark rural area with no street lighting might nevertheless leave her bedroom light on at night and sleep with high LAN, and a woman in a city centre might use shutters or blinds to keep out external light and hence sleep in total darkness. A potential limitation of our study is that it uses self-reported LAN exposure information. However, since this information was ascertained before breast cancer occurrence, it should not have biased the results. Misclassification would be likely to have diluted any true relation, but the lack of any sign of raised risk does not suggest a relationship.

In conclusion, we found no evidence of an association between LAN exposure and raised risk of breast cancer in this large UK-based cohort study. Although our findings raise the possibility of a protective effect in pre-menopausal women who reported night waking with exposure to light at age 20, this was a subset analysis with modest statistical significance, based on relatively few breast cancer cases, has not been reported elsewhere, and has no plausible mechanism, so cannot be taken as strong evidence unless confirmed independently.

## Figures and Tables

**Table 1:** Characteristics of Generations Study participants included in the analysis^a^

**Characteristic**	**No.**	**(%)**
**Year of birth**		
1908–1949	29 228	(27.6)
1950–1959	25 516	(24.1)
1960–1969	24 054	(22.7)
1970–1996	27 068	(25.6)
**Age at recruitment (years)**		
<20	1178	(1.1)
20–39	33 482	(31.6)
40–49	24 340	(23.0)
50–59	26 922	(25.4)
≥60	19 944	(18.8)
**Ethnicity**		
White	104 595	(98.8)
Other or not stated	1271	(1.2)
**Socioeconomic status at recruitment**^b^		
1 (highest)	46 168	(43.6)
2	10 224	(9.7)
3	31 732	(30.0)
4	11 749	(11.1)
5 (lowest)	4778	(4.5)
Not classifiable	1215	(1.2)
**Menopausal status at recruitment**		
Post-menopausal	41 181	(38.9)
Pre-menopausal	59 499	(56.2)
Never had periods	34	(0.0)
Not known	5152	(4.9)
**Light at night**^c^		
At recruitment		
Low	22 155	(20.9)
Med	51 889	(49.0)
High	31 822	(30.1)
At age 20 years		
Low	18 750	(17.7)
Med	50 116	(47.3)
High	34 106	(32.0)
Not applicable^d^	1178	(1.1)
Not known	1716	(1.6)
**History of night shift work during preceding 10 years**		
No	87 935	(83.1)
Yes	17 931	(16.9)
**Waking at night and exposed to light**^e^		
At recruitment		
No	58 818	(55.6)
Yes	38 710	(36.6)
Not known	8338	(7.9)
At age 20 years		
No	82 936	(78.3)
Yes	10 183	(9.6)
Not applicable^d^	1178	(1.1)
Not known	11 569	(10.9)
**Non-peak sleep**^f^		
No	105 116	(99.3)
Yes	750	(0.7)
**Sleep duration per night (hours)**		
<7	19 288	(18.2)
⩾7	85 807	(81.1)
Not known	771	(0.7)
Total participants	105 866	(100.0)

Abbreviations: SD=standard deviation.

a At recruitment unless otherwise stated.

b Based on place of residence ACORN score (Solutions CI, 2002).

c Low: ‘Too dark to see your hand, or you wear a mask’ Med: ‘Light enough to see your hand in front of you, but not see across the room’ High: ‘Light enough to see across the room, but not read’+‘Light enough to read’.

d Aged <20 at recruitment.

e Wake and put the lights on or go into a bright room.

f Going to sleep at or after 0200 hours or rising for the day at or before 0100 hours (Davis *et al*, 2001).

**Table 2:** Light at night at recruitment and risk of breast cancer, by menopausal status and oestrogen-receptor status of breast cancer

				**Oestrogen-receptor status of breast cancer**					
	**All breast cancers**			**Positive**			**Negative**		
**LAN level**^a^	**No. cases**	**HR (95% CI)**^b^	***P*****-value**	**No. cases**	**HR (95% CI)**^b^	***P*****-value**	**No. cases**	**HR (95% CI)**^b^	***P*****-value**
**Total (Pyrs=640 832)**									
Low	416	1.00		330	1.00		54	1.00	
Med	847	1.00 (0.89–1.12)	0.97	661	0.99 (0.87–1.13)	0.88	134	1.20 (0.88–1.65)	0.26
High	512	1.01 (0.88–1.15)	0.92	391	0.98 (0.84–1.14)	0.78	77	1.16 (0.82–1.65)	0.40
**Pre-menopausal breast cancer (Pyrs=373 323)**									
Low	145	1.00		115	1.00		19	1.00	
Med	326	0.91 (0.74–1.10)	0.33	250	0.89 (0.71–1.11)	0.30	53	1.09 (0.64–1.84)	0.75
High	219	1.00 (0.81–1.24)	1.00	165	0.97 (0.76–1.24)	0.82	31	1.04 (0.59–1.85)	0.89
**Post-menopausal breast cancer (Pyrs=267 509)**									
Low	271	1.00		215	1.00		35	1.00	
Med	521	1.05 (0.91–1.22)	0.48	411	1.05 (0.89–1.24)	0.55	81	1.26 (0.84–1.87)	0.26
High	293	1.00 (0.85–1.18)	1.00	226	0.97 (0.81–1.17)	0.77	46	1.23 (0.79–1.92)	0.36

Abbreviations: CI=confidence interval; HR=hazard ratio; LAN=light at night; Pyrs=person-years of follow-up.

a Low: ‘Too dark to see your hand, or you wear a mask’ Med: ‘Light enough to see your hand in front of you, but not see across the room’ High: ‘Light enough to see across the room, but not read’+‘Light enough to read’.

b Hazard ratios estimated using Cox proportional hazards regression with attained age as time scale, adjusted for: year of birth, history of benign breast disease, breast cancer in a first-degree relative, socioeconomic score, age at menarche, age at first birth, parity, duration of breastfeeding, oral contraceptive use, hormone replacement therapy use, menopausal status and age at menopause where applicable, pre-menopausal and post-menopausal body mass index, alcohol consumption, smoking and physical activity level.

**Table 3:** Light at night at age 20 and risk of breast cancer, by menopausal status and oestrogen-receptor status of breast cancer

				**Oestrogen-receptor status of breast cancer**					
	**All breast cancers**			**Positive**			**Negative**		
**LAN level**^a^	**No. cases**	**HR (95% CI)**^b^	***P*****-value**	**No. cases**	**HR (95% CI)**^b^	***P*****-value**	**No. cases**	**HR (95% CI)**^b^	***P*****-value**
**Total (Pyrs=624 049)**									
Low	452	1.00		269	1.00		57	1.00	
Med	846	1.02 (0.90–1.16)	0.76	674	1.07 (0.93–1.24)	0.34	118	0.86 (0.63–1.18)	0.36
High	540	1.00 (0.88–1.15)	0.97	409	1.00 (0.86–1.17)	0.96	84	0.94 (0.67–1.32)	0.73
**Pre-menopausal breast cancer (Pyrs=364 008)**									
Low	125	1.00		93	1.00		22	1.00	
Med	321	0.88 (0.71–1.08)	0.21	247	0.92 (0.72–1.16)	0.48	49	0.73 (0.44–1.21)	0.22
High	238	0.91 (0.73–1.13)	0.40	185	0.97 (0.76–1.25)	0.81	31	0.64 (0.37–1.11)	0.11
**Post-menopausal breast cancer (Pyrs=260 042)**									
Low	227	1.00		176	1.00		35	1.00	
Med	525	1.11 (0.95–1.29)	0.20	427	1.17 (0.98–1.39)	0.09	69	0.93 (0.62–1.39)	0.72
High	302	1.04 (0.88–1.24)	0.63	224	1.00 (0.821.22)	0.99	53	1.17 (0.76–1.80)	0.47

Abbreviations: CI=confidence interval; HR=hazard ratio; LAN=light at night; Pyrs=person-years of follow-up.

a Low: ‘Too dark to see your hand, or you wear a mask’ Med: ‘Light enough to see your hand in front of you, but not see across the room’ High: ‘Light enough to see across the room, but not read’+‘Light enough to read’.

b Hazard ratios estimated using Cox proportional hazards regression with attained age as time scale, adjusted for: year of birth, history of benign breast disease, breast cancer in a first-degree relative, socioeconomic score, age at menarche, age at first birth, parity, duration of breastfeeding, oral contraceptive use, hormone replacement therapy use, menopausal status and age at menopause where applicable, pre-menopausal and post-menopausal body mass index, alcohol consumption, smoking and physical activity level.

**Table 4:** Night waking with exposure to light^a^ in the year before recruitment and risk of breast cancer, by menopausal status and oestrogen receptor status of breast cancer

				**Oestrogen-receptor status of breast cancer**					
	**All breast cancers**			**Positive**			**Negative**		
**Night waking**	**No. cases**	**HR (95% CI)**^b^	***P*****-value**	**No. cases**	**HR (95% CI)**^b^	***P*****-value**	**No. cases**	**HR (95% CI)**^b^	***P*****-value**
**Total (Pyrs=640 832)**									
No	939	1.00		729	1.00		142	1.00	
Yes	674	1.01 (0.92–1.12)	0.82	524	1.01 (0.90–1.13)	0.90	100	1.01 (0.78–1.32)	0.91
N/k	162	1.00 (0.84–1.18)	0.99	129	1.01 (0.83–1.22)	0.93	23	1.00 (0.64–1.56)	0.99
**Pre-menopausal breast cancer (Pyrs=373 323)**									
No	412	1.00		317	1.00		59	1.00	
Yes	247	1.10 (0.93–1.29)	0.26	188	1.09 (0.91–1.31)	0.35	39	1.24 (0.82–1.86)	0.31
N/k	31	0.63 (0.43–0.91)	0.01	25	0.64 (0.43–0.97)	0.04	5	0.81 (0.32–2.03)	0.65
**Post-menopausal breast cancer (Pyrs=267 509)**									
No	527	1.00		412	1.00		83	1.00	
Yes	427	0.96 (0.85–1.10)	0.58	336	0.96 (0.83–1.11)	0.62	61	0.90 (0.64–1.26)	0.54
N/k	131	1.15 (0.95–1.40)	0.16	104	1.16 (0.93–1.45)	0.18	18	1.03 (0.62–1.74)	0.90

Abbreviations: CI=confidence interval; HR=hazard ratio; Pyrs=person-years of follow-up.

a Wake and put the lights on or go into a bright room.

b Hazard ratios estimated using Cox proportional hazards regression with attained age as time scale, adjusted for: light at night in the year before recruitment, year of birth, history of benign breast disease, breast cancer in a first-degree relative, socioeconomic score, age at menarche, age at first birth, parity, duration of breastfeeding, oral contraceptive use, hormone replacement therapy use, menopausal status and age at menopause where applicable, pre-menopausal and post-menopausal body mass index, alcohol consumption, smoking and physical activity level.

**Table 5:** Night waking with exposure to light^a^ at age 20 and risk of breast cancer, by menopausal status and oestrogen receptor status of breast cancer

				**Oestrogen-receptor status of breast cancer**					
	**All breast cancers**			**Positive**			**Negative**		
**Night waking**	**No. cases**	**HR (95% CI)**^b^	***P*****-value**	**No. cases**	**HR (95% CI)**^b^	***P*****-value**	**No. cases**	**HR (95% CI)**^b^	***P*****-value**
**Total (Pyrs=640 832)**									
No	1450	1.00		1130	1.00		220	1.00	
Yes	103	0.85 (0.70–1.04)	0.12	77	0.82 (0.65–1.04)	0.10	15	0.82 (0.49–1.40)	0.47
N/k	222	0.91 (0.78–1.05)	0.20	175	0.90 (0.77–1.07)	0.23	30	0.85 (0.57–1.26)	0.41
**Pre-menopausal breast cancer (Pyrs=373 323)**									
No	593	1.00		457	1.00		88	1.00	
Yes	50	0.74 (0.55–0.99)	0.04	36	0.69 (0.49–0.97)	0.03	9	0.91 (0.45–1.82)	0.79
N/k	47	0.65 (0.48–0.87)	0.01	37	0.65 (0.46–0.91)	0.01	6	0.64 (0.28–1.49)	0.30
**Post-menopausal breast cancer (Pyrs=267 509)**									
No	857	1.00		673	1.00		132	1.00	
Yes	53	0.96 (0.73–1.27)	0.80	41	0.95 (0.69–1.30)	0.74	6	0.72 (0.32–1.63)	0.43
N/k	175	1.02 (0.86–1.21)	0.80	138	1.02 (0.84–1.23)	0.85	24	0.93 (0.60–1.46)	0.76

Abbreviations: CI=confidence interval; HR=hazard ratio; Pyrs=person-years of follow-up.

a Wake and put the lights on or go into a bright room.

b Hazard ratios estimated using Cox proportional hazards regression with attained age as time scale, adjusted for: light at night at age 20, light at night in the year before recruitment, year of birth, history of benign breast disease, breast cancer in a first-degree relative, socioeconomic score, age at menarche, age at first birth, parity, duration of breastfeeding, oral contraceptive use, hormone replacement therapy use, menopausal status and age at menopause where applicable, pre-menopausal and post-menopausal body mass index, alcohol consumption, smoking and physical activity level.
