# Supplementary material for: Domestic light at night and breast cancer risk: a prospective analysis of 105 000 UK women in the Generations Study
Source: Br J Cancer. 2018 Jan 23;118(4):600–6. doi: 10.1038/bjc.2017.359 (PMC5830585; doi:10.1038/bjc.2017.359)
Supplement: Supplementary Table 1 [file bjc2017359x1.docx]

| **Supplementary Table 1 Characteristics of breast cancer cases included in the analysis** |
| --- |

| **Characteristic** |  | **Breast cancer cases** | |
| --- | --- | --- | --- |
|  |  | **No.** | **%** |
| **Age at recruitment (years)** | <20 | 0 | 0% |
|  | 20-39 | 164 | 9% |
|  | 40-49 | 455 | 26% |
|  | 50-59 | 600 | 34% |
|  | ≥60 | 556 | 31% |
|  |  |  |  |
| **Socioeconomic status at recruitment** | 1 (highest) | 904 | 51% |
|  | 2 | 141 | 8% |
|  | 3 | 514 | 29% |
|  | 4 | 153 | 9% |
|  | 5 (lowest) | 48 | 3% |
|  | Not classifiable | 15 | 1% |
|  |  |  |  |
| **Menopausal status at recruitment** | Post-menopausal | 1028 | 58% |
|  | Pre-menopausal | 650 | 37% |
|  | Never had periods | 1 | <1% |
|  | Not known | 96 | 5% |
|  |  |  |  |
| **Menopausal status at breast cancer diagnosis** | Post-menopausal | 1085 | 61% |
|  | Pre-menopausal | 690 | 39% |
|  |  |  |  |
| **Age at breast cancer diagnosis** | 20-29 | 7 | <1% |
|  | 30-39 | 86 | 5% |
|  | 40-49 | 341 | 19% |
|  | 50-59 | 530 | 30% |
|  | 60-69 | 631 | 36% |
|  | 70-79 | 161 | 9% |
|  | ≥80 | 19 | 1% |
|  |  |  |  |
| **Confirmation of breast cancer** | Yes^a^ | 1773 | >99% |
|  | No^b^ | 2 | <1% |
|  |  |  |  |
| **Invasive status** | Invasive | 1503 | 85% |
|  | *In situ* | 272 | 15% |
|  |  |  |  |
| **Histological type** | Ductal | 1366 | 77% |
|  | Lobular | 274 | 15% |
|  | Other | 42 | 2% |
|  | Not Known | 93 | 5% |
|  |  |  |  |
| **Oestrogen-receptor (ER) status** | Positive | 1382 | 78% |
|  | Negative | 265 | 15% |
|  | Not known | 128 | 7% |

Confirmation of breast cancer cases: ^a^ Yes=Confirmation through national cancer registration or medical records; ^b^ No=with reported treatments that may imply self-reported diagnosis of breast cancer is correct
